# Modulatory Effect of Blood LDL Cholesterol on the Association between Cerebral Aβ and Tau Deposition in Older Adults

**DOI:** 10.14283/jpad.2024.131

**Published:** 2024-07-02

**Authors:** S. M. Han, M. S. Byun, D. Yi, J. H. Jung, N. Kong, Y. Y. Chang, M. Keum, G. J. Jung, J.-Y. Lee, Y.-S. Lee, Y. K. Kim, K. M. Kang, C.-H. Sohn, Dong Young Lee, KBASE Research Group

**Affiliations:** 1 Department of Psychiatry, Seoul National University College of Medicine, Seoul, Republic of Korea; 2 Department of Neuropsychiatry, Seoul National University Hospital, 101, Daehak-ro, Jongno-gu, Seoul, Republic of Korea 03080; 3 Institute of Human Behavioral Medicine, Medical Research Center, Seoul National University, Seoul, Republic of Korea; 4 Department of Neuropsychiatry, Chungbuk National University Hospital, Cheongju, Republic of Korea; 5 Department of Psychiatry, Keimyung University Dongsan Hospital, Daegu, Republic of Korea; 6 Department of Psychiatry, Sanggye Paik Hospital, College of Medicine, Inje University, Seoul, Republic of Korea; 7 Department of Neuropsychiatry, Hallym University Dongtan Sacred Heart Hospital, Hwaseong, Republic of Korea; 8 Department of Neuropsychiatry, SMG-SNU Boramae Medical Center, Seoul, Republic of Korea; 9 Department of Nuclear Medicine, Seoul National University Hospital, Seoul, Republic of Korea; 10 Department of Nuclear Medecine, SMG-SNU Boramae Medical Center, Seoul, Republic of Korea; 11 Department of Radiology, Seoul National University Hospital, Seoul, Republic of Korea; 12 Interdisciplinary program of cognitive science, Seoul National University College of Humanities, Seoul, Republic of Korea

**Keywords:** Low-density lipoprotein cholesterol, beta-amyloid, tau, Alzheimer’s disease, neurodegeneration

## Abstract

**Background:**

This study investigates the synergistic relationship between blood low-density lipoprotein cholesterol (LDL-C) and cerebral beta-amyloid (Aβ) in relation to tau deposition, a key factor in the pathology of Alzheimer’s disease (AD), in older adults across a diverse cognitive spectrum.

**Objectives:**

To examine whether higher levels of LDL-C in the blood moderate the association of cerebral Aβ with tau deposition in older adults, including those with normal cognition, mild cognitive impairment, and Alzheimer’s disease dementia.

**Design:**

Cross-sectional design. Setting: The study was conducted as a part of a prospective cohort study. All assessments were done at the Seoul National University Hospital, Seoul, South Korea. Participants: A total of 136 older adults (aged 60–85 years) with normal cognition, mild cognitive impairment or Alzheimer’s disease (AD) dementia were included.

**Measurements:**

Serum lipid measurements, [11C] Pittsburgh Compound B-positron emission tomography (PET), [18F] AV-1451 PET, and magnetic resonance imaging were performed on all participants.

**Results:**

There was a significant Aβ × LDL-C interaction effect on tau deposition indicating a synergistic moderation effect of LDL-C on the relationship between Aβ and tau deposition. Subsequent subgroup analysis showed that the positive association between Aβ and tau deposition was stronger in higher LDL-C group than in lower LDL-C group. In contrast, other lipids, such as total cholesterol, high-density lipoprotein cholesterol, and triglycerides, did not show a similar moderation effect on the relationship between Aβ deposition and tau deposition.

**Conclusion:**

Our findings suggest that blood LDL-C synergistically enhances the influence of Aβ deposition on tau pathology, emphasizing the need for greater attention to the role of LDL-C in AD progression.

**Electronic Supplementary Material:**

Supplementary material is available in the online version of this article at 10.14283/jpad.2024.131.

## Introduction

**H**ypercholesterolemia in midlife and early late-life has been proposed as a potential risk factor for dementia (1–3). Higher blood low-density lipoprotein cholesterol (LDL-C) levels, in particular, have been repeatedly related to Alzheimer’s disease (AD) and related cognitive decline (4, 5).

Nevertheless, the underlying neuropathological links in the relationship between increased blood LDL-C and AD-related cognitive impairment are not clearly understood. A cell culture study suggested that LDL-C may increase not only beta-amyloid protein (Aβ) but also phosphorylated tau protein (6). Other preclinical studies also showed that apolipoprotein B containing LDL-C increased the accumulation of phosphorylated tau in brain by inhibiting the activity of cathepsin D in autophagotic lysosomes (7). Lysosomal enzyme cathepsin D is known to degrade tau protein (8). Given the well-established relationship between Aβ and tau deposition in AD (9, 10) such influence of LDL-C on tau protein raises the possibility that LDL-C level may synergistically increase tau deposition with Aβ pathology. Although a couple of human studies reported that serum LDL-C levels were associated with an increase of brain Aβ accumulation (11, 12), and cerebrospinal fluid (CSF) levels of apolipoprotein B (APOB), the primary apolipoprotein of LDL-C, were correlated with CSF tau levels or brain tau deposition (13) little is known about the synergistic interaction between blood LDL-C and Aβ for tau deposition in the human brain.

In this context, we aimed to test the hypothesis that blood LDL-C moderates the association between cerebral Aβ and tau deposition in older adults with a diverse cognitive spectrum including cognitively normal (CN), mild cognitive impairment (MCI), and AD dementia. We also explored the associations of various blood lipids, including total cholesterol (TC), LDL-C, high-density lipoprotein cholesterol (HDL-C), and triglyceride (TG) with in vivo AD pathologies and cerebrovascular injury as measured by white matter hyperintensities (WMHs).

## Methods

### Participants

The current study was conducted for the part of older individuals who participated in the Korean Brain Aging Study for Early Diagnosis and Prediction of Alzheimer’s Disease (KBASE), a prospective cohort study which begun in 2014. The main purposes of the KBASE are to find new AD biomarkers and investigate how different life events and physical changes affect AD-related brain alterations (14). Participants were recruited from four sites in Seoul, Republic of Korea. Potential study candidates who attended memory clinics at two university hospitals in Seoul or participated in dementia screening programs at two public dementia prevention and management centers were informed about the study and invited to be evaluated for eligibility. Furthermore, community volunteers were recruited through personal referrals, posters and brochures, and online advertisements. The present study finally included 136 older adults (aged 55 to 90) who had undergone tau-PET scans as of August 22, 2018. They consisted of 69 cognitively normal (CN), 32 mild cognitive impairment (MCI), and 35 Alzheimer’s disease (AD) dementia individuals. Those without dementia and MCI diagnosis and with Clinical Dementia Rating (CDR)15 score of 0 were classified as CN. All MCI patients satisfied the commonly accepted criteria for amnestic MCI16 which encompass (1) subjective or informant-corroborated memory complaints; (2) demonstrated objective memory deficits; (3) intact overall cognitive function; (4) independence in daily activities; and (5) no dementia. For the second criterion, a z-score of 1.0 in at least one of the four episodic memory tests was applied. AD dementia was diagnosed according to the fourth edition of the Diagnostic and Statistical Manual of Mental Disorders17 and the National Institute on Aging and Alzheimer’s Association guidelines for probable AD.18 All participants with AD dementia also had a global CDR score of either 0.5 or 1. Exclusion criteria included a major psychiatric disorder and significant neurological or medical illnesses that could alter mental status. The use of investigational medications, significant vision or hearing impairments, illiteracy, severe communication or behavioral problems that might hinder clinical assessments or brain imaging were additional exclusion criteria. The study procedure received approval from the institutional review boards of Seoul National University Hospital (C-1401-027-547) and SNU-SMG Boramae Medical Center (26-2015-60), and adhered to the latest version of the Declaration of Helsinki. Participants or their authorized representatives gave their written consent after receiving comprehensive information about the study.

### Clinical assessments

Using the KBASE assessment procedure (14), which included and enhanced the Korean edition of the Consortium to Establish a Registry for Alzheimer’s Disease assessment package (CERAD-K), comprehensive clinical data were gathered from each participant (19). The presence of dyslipidemia and additional vascular risk factors including diabetes mellitus, hypertension, transient ischemic attack, coronary heart disease, and stroke was systematically evaluated based on information gathered by skilled nurses through systematic interviews with participants and their informants and medical record reviews. Participants were deemed to have dyslipidemia if they had received a dyslipidemia diagnosis at a medical facility or were already receiving medication for the condition at the time of enrollment. The total number of vascular risk factors other than dyslipidemia was regarded as a vascular risk score reflecting the burden of vascular risk aside from dyslipidemia (VRSnoDLP) (20).

### Measurement of cerebral Aβ deposition

Simultaneous acquisition of three-dimensional [11C] Pittsburgh compound B (PiB)-positron emission tomography (PET) and T1-weighted MRI images was performed using a 3.0T Biograph mMR (PET-MR) scanner (Siemens; Washington DC, WC, USA) for all participants. The methods for PiB-PET image acquisition and preprocessing were detailed in a previous report.10 By employing automated anatomical labeling and a region-combining method (21), regions of interests (ROIs) were established to examine PiB retention in various brain regions, including the frontal, lateral temporal, lateral parietal, and posterior cingulate-precuneus areas. To calculate the standardized uptake value ratio (SUVR) for each ROI, the average voxel value within each ROI was divided by the corresponding mean cerebellar uptake value. A global Aβ deposition was calculated by dividing the average voxel value in the global cortical ROI, which consists of the 4 ROIs, by the mean cerebellar uptake value (21).

### Measurement of tau deposition

[18F] AV-1451 PET scans were obtained for all subjects using a Siemens Biograph True Point 40 PET/CT scanner. [18F] AV-1451 PET imaging was performed on average 2.6 years (SD = 0.3) after the baseline assessment including clinical evaluations, other brain imaging including PiB-PET and blood sampling. The detailed methods for AV-1451 PET image acquisition and preprocessing were described in our earlier report.10 The AV-1451 SUVR value of «AD-signature regions» of tau deposition, which is compared of a size-weighted mean of partial volume-corrected uptake in the middle temporal, inferior temporal, fusiform, parahippocampal, entorhinal, and amygdala ROIs (22), was used as an outcome variable for global tau deposition.

### Measurement of WMHs

Fluid-attenuated inversion recovery images obtained with the same MRI scanner were used to estimate the volume of the cerebral WMHs. We used an automated process that had already been validated (23) with two changes. First, as it was more appropriate for our data, a threshold value of 70 rather than 65 from the original reference was applied. Second, because individuals with acute cerebral infarction were not included, diffusion-weighted imaging was not used in the process.

### Measurements of lipid profiles

Participants’ blood was drawn in the morning (8–9 a.m.) after an overnight fast and kept in a serum separator tube (Becton, Dickinson and Co., Franklin Lakes, NJ, USA) At room temperature, the tubes were centrifuged at 1300 x g for 10 minutes. The serum supernatants were then collected and stored at −80°C. Serum levels of TC, LDL-C, HDL-C, and TG were measured at Seoul Clinical Laboratories (SCL) with a colorimetric technique and an ADVIA 1800 Auto Analyzer (Siemens, USA).

### APOE genotyping

Genomic DNA was isolated from venous blood samples. Apolipoprotein E (APOE) genotyping followed the protocol outlined by Wenham et al (24).

### Statistical analysis

We initially explored the association of each lipid with AD biomarkers and WMH volume through partial correlation analysis using age, education, gender, APOE ε4 positivity and VRSnoDLP as covariates. To test the main hypothesis that blood LDL-C moderates the association between cerebral Aβ and tau deposition, we analyzed the multiple linear regression model including Aβ deposition × LDL-C interaction term as well as Aβ deposition and LDL-C as independent variables with tau deposition as the dependent variable and age, gender, education, APOE ε4 positivity, and VRSnoDLP as covariates. When the interaction term was significant, we performed two further analyses for LDL-C subgroups, separately. First, The participants were divided into lower (<116 mg/dL) and higher (≥116 mg/dL) LDL-C subgroups according to the 2019 European Society of Cardiology (ESC) / European Atherosclerosis Society (EAS) guideline for the management of dyslipidemia (25). Next, we divided the subjects into three groups based on their LDL-C levels, and conducted same analysis for each group: the low-level group (n=45), the mid-level group (n=45), and the high-level group (n=46). We additionally performed exploratory analyses for the moderating effect of other lipids (TC, HDL-C, and TG) on the relationships between Aβ and tau deposition using a similar regression model. All analyses were conducted using IBM SPSS Statistics 25 software (IBM Corporation, Armonk, NY, USA) and a threshold of p < 0.05 was set to determine statistical significance for all the analyses.

### Data availability

The data can be accessed through the KBASE research group’s independent data sharing committee upon a reasonable request. Email requests for data access can be sent to the KBASE group’s administrative coordinator (kbasecohort@gmail.com).

## Results

### Characteristics of Participants

Demographic and medical attributes of the participants are presented in Table 1. As shown in Table 1, lower (<116 mg/dL) LDL-C group had more common history of hypertension and dyslipidemia, higher VRSnoDLP, and more frequent use of statin, but lower LDL-C and TC, than higher LDL-C (≥116 mg/dL) group.

**Table 1:** Characteristics of participants

**Variables**	**All patients (N=136)**	**LDL-C <116 mg/dL (N=88)**	**LDL-C ≥116 mg/dL (N=48)**	**P value**
Clinical characteristics				
Age (years)	73.4 (7.7)	74.5 (7.2)	71.3 (8.1)	0.019 ^a^
Females	86 (63.2%)	54 (61.4%)	32 (66.7%)	0.540 ^b^
Educational level (years)	10.9 (4.8)	11.07 (5.0)	10.58 (4.6)	0.578 ^a^
APOE ε4 carriers	41 (30.1%)	29 (33.0%)	12 (25.0%)	0.334 ^b^
CN/MCI/AD dementia	69/32/35 (50.7%/23.5%/25.7%)	42/22/24 (47.7%/25.0%/27.3%)	27/10/11 (56.3%/20.8%/22.9%)	0.637 ^b^
Hypertension	73 (53.7%)	55 (62.5%)	18 (37.5%)	0.005 ^b^
DM	35 (25.7%)	27 (30.7%)	8 (16.7%)	0.074 ^b^
Coronary heart disease	10 (7.4%)	9 (10.2%)	1 (2.1%)	0.082 ^b^
Stroke	1 (0.7%)	1 (1.1%)	0 (0%)	0.459 ^b^
TIA	0 (0%)	0 (0%)	0 (0%)	-
Dyslipidemia	58 (42.6%)	46 (52.3%)	12 (25%)	0.002 ^b^
VRSnoDLP	0.88 (0.8)	1.05 (0.8)	0.56 (0.8)	0.001 ^b^
LDL-C (mg/dL)	106.9 (36.8)	85.6 (19.8)	146.1 (27.3)	0.000 ^a^
TC (mg/dL)	178.5 (40.3)	156.9 (25.3)	218.2 (31.7)	0.000 ^a^
HDL-C (mg/dL)	53.5 (13.9)	52.5 (14.1)	55.3 (13.5)	0.273 ^a^
TG (mg/dL)	124.6 (67.7)	123.0 (75.1)	127.7 (52.2)	0.698 ^a^
Statin use	40 (29.4%)	32 (36.4%)	8 (16.7%)	0.016 ^b^
Global Aβ deposition	1.47 (0.5)	1.51 (0.5)	1.40 (0.5)	0.239 ^a^
Tau deposition	1.73 (0.9)	1.73 (0.8)	1.74 (1.1)	0.970 ^a^
WMHs (volume, cm^3^)	7.49 (9.3)	8.28 (10.6)	6.06 (6.1)	0.184 ^a^

Notes: Data are presented as mean (SD) or n (%). a. By Independent samples t-test; b. By Chi-squared test; Abbreviations: Aβ, beta-amyloid; AD, Alzheimer’s disease; APOE, apolipoprotein E; CN, cognitively normal; DM, diabetes mellitus; HDL-C, high density lipoprotein cholesterol; LDL-C, low-density lipoprotein cholesterol; MCI, mild cognitive impairment; TC, total cholesterol; TG, triglyceride; TIA, transient ischemic attack; VRSnoDLP, vascular risk score reflecting vascular risk burden other than dyslipidemia; WMHs, white matter hyperintensities.

### Partial correlation between serum lipids and Aβ deposition, tau deposition and WMH volume

We did not find any association between serum lipids and Aβ or tau deposition (Table 2). There was a significant but weak relationship between LDL-C and WMH volume, while other lipids did not relate with the volume.

**Table 2:** Partial correlation between serum lipids and Aβ deposition, tau deposition and WMH volume

		**LDL-C**	**TC**	**HDL-C**	**TG**
Aβ deposition	correlation	−0.001	−0.038	0.003	−0.058
	P value	0.994	0.668	0.972	0.513
Tau deposition	correlation	0.070	0.045	0.042	−0.063
	P value	0.426	0.610	0.636	0.479
WMH volume	correlation	−0.180	−0.048	0.142	0.022
	P value	0.041	0.591	0.107	0.800

Notes: Partial correlation model included age, gender, education, APOE ε4 positivity, and VRSnoDLP as covariates. Abbreviations: Aβ, Amyloid beta; APOE, apolipoprotein E; HDL-C, high density lipoprotein cholesterol; LDL-C, low-density lipoprotein cholesterol; TC, total cholesterol; TG, triglyceride; VRSnoDLP, vascular risk score reflecting vascular risk burden other than dyslipidemia; WMH, white matter hyperintensity.

### Moderation of LDL-C for the relationship between Aβ deposition and tau deposition

**Figure 1:**
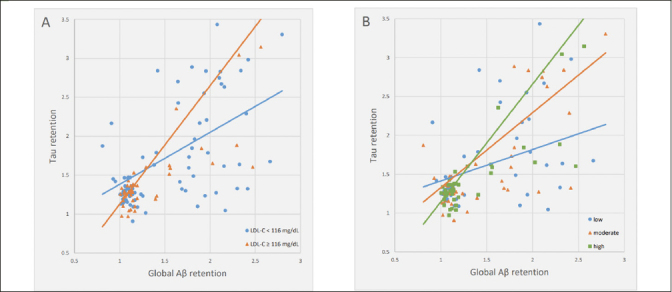
Multiple linear regression plots showing moderating effects of LDL-C on the relationships between Aβ and tau deposition Note: For the purpose of demonstration, participants were divided into (A) two and (B) three LDL-C subgroups. Multiple linear regression model included Aβ, LDL-cholesterol subgroup, and their interaction term as independent variables; tau retention as dependent variable; and age, gender, education, APOE ε4 positivity, and VRSnoDLP as covariates. Statistical significance was observed with the interaction term between Aβ deposition and LDL-C (p < 0.05), as detailed in the manuscript. Abbreviations: Aβ, beta-amyloid; APOE, apolipoprotein; LDL-C, low density lipoprotein cholesterol; VRSnoDLP, vascular risk score reflecting vascular risk burden other than dyslipidemia.

There was a significant Aβ deposition × LDL-C interaction effect on tau deposition, indicating the moderation effect of LDL-C on the relationship between Aβ and tau deposition (Table 3). Sensitivity analyses that controlled for statin use or the time gap between PiB PET and AV-1451 PET as an additional covariate revealed similar results (eTable 1 and eTable 2, respectively). Subsequent subgroup analyses demonstrated that while a significant association between Aβ and tau deposition was observed in both LDL-C subgroups, the association was stronger in higher LDL-C group (≥116 mg/dL) than in lower LDL-C group (<116 mg/dL) (Table 4 and Figure 1A). When additional subgroup analyses were performed for the three tertile groups of LDL-C, the degree of association between Aβ and tau deposition gradually increased from low tertile group to high tertile one (Table 4 and Figure 1B). For the purpose of demonstration, we also provided a figure (eFigure 1) that includes regression lines for the top 50% LDL-C and the bottom 50% LDL-C groups.

**Table 3:** Multiple linear regression analysis for the interaction between Aβ deposition and LDL-C for tau deposition

	**Coefficients**			**t**	**P value**
	**B**	**SE**	**β**		
Dependent variable: Tau deposition^a^					
Global Aβ deposition	0.153	0.376	0.086	0.407	0.696
LDL-C	−0.009	0.005	−0.360	−1.612	0.112
Global Aβ deposition x LDL-C	0.006	0.003	0.586	1.990	0.046

Notes: Multiple linear regression model included age, gender, education, APOE ε4 positivity, and VRSnoDLP as covariates. a. F for the model=8.814; adjusted R2=0.321. Abbreviations: Aβ, beta-amyloid; LDL-C, low density lipoprotein cholesterol; APOE, apolipoprotein; VRSnoDLP, vascular risk score reflecting vascular risk burden other than dyslipidemia.

**Table 4:** Multiple linear regression analysis for the relationship between Aβ and tau deposition in each LDL-cholesterol subgroup

		**Coefficients**			**t**	**R**^2^	**P value**
		**B**	**SE**	**β**			
Dependent variable: Tau deposition							
Global Aβ deposition	LDL-C <116 mg/dL (n=88)	0.573	0.171	0.369	3.339	0.278	0.001
	LDL-C ≥116 mg/dL (n=48)	1.457	0.296	0.664	4.927	0.500	0.000
Global Aβ deposition	LDL-C low-level (n=45)	0.447	0.190	0.375	2.353	0.306	0.024
	LDL-C mid-level (n=45)	0.775	0.303	0.424	–0.405	0.319	0.015
	LDL-C high-level (n=46)	1.377	0.333	0.629	4.136	0.507	0.000

Notes: Multiple linear regression models included age, gender, education, APOE ε4 positivity, and VRSnoDLP as covariates. Abbreviations: Aβ, beta-amyloid; LDL-C, Low density lipoprotein cholesterol; APOE, apolipoprotein; VRSnoDLP, vascular risk score reflecting vascular risk burden other than dyslipidemia.

### Exploratory analyses for the moderation of other lipids

Any other lipids did not have any significant moderation effect for the relationship between Aβ and tau deposition (eTable 3).

## Discussion

While there was no direct relationship of serum LDL-C with brain Aβ and tau deposition, we found significant LDL-C × brain Aβ synergistic interaction on tau deposition, which supported our hypothesis that blood LDL-C moderates the association between cerebral Aβ and tau deposition. Subsequent subgroup analyses demonstrated that the positive association between Aβ and tau deposition was stronger at higher LDL-C levels than at lower LDL-C levels. In contrast to LDL-C, none of the other lipids demonstrated a moderating effect on the Aβ-tau association, nor did they show a direct association with Aβ or tau deposition.

The finding that higher LDL-C levels strengthened the association of brain Aβ with tau deposition is generally consistent with previous clinical reports on the relationship between blood LDL-C and AD-related cognitive decline (4, 5). This finding also aligns with a report showing a reduced risk of AD dementia in users of statin drugs that lower blood LDL-C (2, 6). Similarly, the association between statin use and reduced burden of neurofibrillary tangles at autopsy was reported (27). Although it is not easy to provide the exact mechanisms which underlie the moderation effect of LDL-C on the Aβ-tau relationship, some possible explanation can be made. As the brain is the most cholesterol-rich organ in the body, containing about 20% of the body’s total cholesterol (28), changes in cholesterol levels can lead to brain pathology (29). However, the presence of the blood-brain barrier (BBB) prevents blood cholesterol from entering the brain (30). Nevertheless, free radicals, formed under the influence of high blood cholesterol, can destroy the BBB and, as a result, increase brain cholesterol (30, 31). Elevated brain LDL-C levels have been shown to promote neuroinflammatory responses (32). The induced neuroinflammatory response in turn influence tau pathogenesis (33). A study using an animal model reported that intra-cerebral administration of a potent inflammatory substance to myeloid receptor promoted tau hyper-phosphorylation and tangle formation (34). It was also reported that minocycline treatment reduced cortical tau phosphorylation through reduced inflammation in a mouse model of tauopathy (35). Other transgenic AD model study showed that inflammation exacerbates tau pathology by a cyclin-dependent kinase 5-mediated pathway (36). Meanwhile, microglia, an important regulators of neuroinflammation, attaches to amyloid plaques and abundantly release cytokines that induce neuroinflammation, which potentially exacerbate tau pathology in the periphery of the amyloid plaques (37). Given all together, increased blood LDL-C may aggravate tau accumulation closely related with amyloid pathology (9, 10, 38) by elevating brain LDL-C and in turn promoting neuroinflammatory response.

Additionally, recent studies have highlighted the role of apolipoprotein B (APOB), the primary apolipoprotein of LDL-C, in the neurobiology of AD. Several mutations in the APOB gene in familial cases with early onset AD have been identified, independent of the usual culprits (PEN1, PEN2, and APP) (39). Several studies on transgenic mice have shown that APOB can affect tau pathology, providing evidence for the role of APOB in AD (40–42). A recent human study also demonstrated that cerebrospinal fluid (CSF) APOB levels were elevated in AD patients, and correlated with CSF tau and brain tau PET binding in pre-symptomatic individuals, suggesting CSF APOB markedly associates with early tau dysregulation (13). Given the progressive deterioration of the BBB at the MCI and AD stages (40, 41), which allows APOB and LDL-C to enter the brain (13, 39), these findings are particularly relevant to our results regarding the contribution of blood LDL-C, an acolyte of APOB, on brain tau deposition.

In contrast to the moderation effect of LDL-C, other lipids did not have any relationship with Aβ or tau accumulation in our study. Several studies have shown that high TC or low HDL-C levels are associated with clinical diagnosis of AD dementia or cognitive impairment (43, 44). However, direct comparison between the findings of the previous studies and those of the current study is not easy because the considerable differences in study methodology. While we focused on the associations of lipids on in vivo AD pathologies, most previous studies did not measure the pathologies and just investigated the relationship with clinically defined AD dementia or cognitive impairment (43, 44). Approximately 14–32% of clinically defined AD dementia cases (45) and 29–73% of MCI cases did not exhibit Aβ pathology in the brain.46 In addition, many of the previous studies investigated the relationship between lipid levels in midlife and dementia in late-life (43, 44) while we measured lipid levels and in vivo brain pathologies in late-life. A study reported that high midlife TG levels were associated with increased brain Aβ and tau pathology after 20 years (11).

Our exploratory analyses showed that serum LDL-C was positively associated WMH volume, but other lipids were not. This is consistent with the results of other studies: An observational study showed that high blood LDL-C was associated with increased WMHs (47), and another study also reported that LDL-C was related with periventricular WMHs, but other lipids, i.e., TC, HDL-C, and TG, were not (48). Since brain endothelial cells are sensitive to circulating LDL-C levels, impaired vascular responses are induced through oxidative stress and the secretion of inflammatory mediators caused by increased LDL-C (49).

Our result that serum LDL-C has a moderating influence on brain Aβ-tau association in human is new. The present study does, however, include several limitations that need to be taken into account. Firstly, because this research was cross-sectional, no causal connections can be determined by the results. Additional prospective longitudinal research is necessary. Second, it may have been difficult to identify a direct link between higher serum LDL-C levels and each of Aβ and tau deposition because of the limited number of participants (n=48, 34.6%) with unusually high serum LDL-C levels (116mg/dL). Lastly, while amyloid PET and MRI scans were performed at baseline, tau PET scans were conducted on average 2.6 years (standard deviation = 0.3 years) after the baseline visit. The relationship between blood lipids and tau deposition may have been affected by this time gap. However, the results remained consistent when the temporal gap was adjusted as an extra covariate.

Our findings suggest that blood LDL-C synergistically increases tau pathology with Aβ deposition. In terms of AD pathophysiology, more attention need to be paid to the role of LDL-C.

## Electronic supplementary material

Supplementary material, approximately 52 KB.

## Data Availability

*Availability of data and materials:* Data supporting the findings of this study are available from the Korean Brain Aging Study for Early Diagnosis and Prediction of Alzheimer’s Disease (KBASE) research group but are not publicly available. Data access requests can be made by contacting the KBASE group’s administrative coordinator via email (kbasecohort@gmail.com).

## Abbreviations

Aβ: beta-amyloid
AD: Alzheimer’s disease
APOE: apolipoprotein
BBB: blood-brain barrier
CDR: Clinical Dementia Rating
CERAD-K: Consortium to Establish a Registry for Alzheimer’s Disease assessment package
CN: cognitively normal
HDL-C: high-density lipoprotein cholesterol
KBASE: Korean Brain Aging Study for Early Diagnosis and Prediction of Alzheimer’s Disease
LDL-C: low density lipoprotein cholesterol
MCI: mild cognitive impairment
PET: positron emission tomography
PiB: Pittsburgh compound B
ROI: regions of interest
SUVR: standardized uptake value ratio
TC:TG: triglyceride; total cholesterol
VRSnoDLP: vascular risk score reflecting vascular risk burden other than dyslipidemia
WMHs: white matter hyperintensities

## Notes

*Ethics approval and consent to participate:* The study was approved by the Institutional Review Boards of Seoul National University Hospital (IRB No: C-1401-027-547) and SNU-SMG Boramae Medical Center (IRB No: 26-2015-60). All procedures were in accordance with the latest version of the Declaration of Helsinki. Written informed consent was obtained from all individual participants included in the study or their authorized representatives.

## Notes

*Consent for publication:* Not applicable.

## Notes

*Data Statement:* The data has not been previously presented orally or by poster at scientific meetings.
