# Supplementary material for: Modulatory Effect of Blood LDL Cholesterol on the Association between Cerebral Aβ and Tau Deposition in Older Adults
Source: J Prev Alzheimers Dis. 2024 Jul 2;11(6):1767–74. doi: 10.14283/jpad.2024.131 (PMC11573824; doi:10.14283/jpad.2024.131)
Supplement: Supplementary file 1 — Supplementary material, approximately 52 KB. [file 42414_2024_131_MOESM1_ESM.docx]

**Supplementary materials**

| **eTable 1.** Multiple linear regression analysis for the interaction between Aβ deposition and LDL-C for tau deposition controlling statin use as an additional covariate | | | | | | | |
| --- | --- | --- | --- | --- | --- | --- | --- |
|  |  |  | Coefficients | | | t | *P* value |
|  |  |  | B | SE | β |  |  |
| Dependent variable: Tau deposition^a^ | | |  |  |  |  |  |
| Global Aβ deposition | | | 0.149 | 0.378 | 0.084 | 0.393 | 0.695 |
| LDL-C | | | -0.009 | 0.005 | -0.363 | -1.613 | 0.109 |
| Global Aβ deposition x LDL-C | | | 0.006 | 0.003 | 0.589 | 1.988 | 0.049 |
| **Notes:** Multiple linear regression model included age, gender, education, APOE ε4 positivity, VRS_noDLP_, and statin use as covariates.  ^a^ F for the model=7.776; adjusted R^2^=0.316. **Abbreviations:** Aβ, beta-amyloid; LDL-C, low density lipoprotein cholesterol; APOE, apolipoprotein; VRS_noDLP_, vascular risk score reflecting vascular risk burden other than dyslipidemia. | | | | | | | |

| **eTable 2.** Multiple linear regression analysis for the interaction between Aβ deposition and LDL-C for tau deposition with time gap as an additional covariate | | | | | | | |
| --- | --- | --- | --- | --- | --- | --- | --- |
|  |  |  | Coefficients | | | t | *P* value |
|  |  |  | B | SE | β |  |  |
| Dependent variable: Tau deposition^a^ | | |  |  |  |  |  |
| Global Aβ deposition | | | 0.141 | 0.381 | 0.079 | 0.369 | 0.713 |
| LDL-C | | | -0.009 | 0.005 | -0.369 | -1.621 | 0.108 |
| Global Aβ deposition x LDL-C | | | 0.006 | 0.003 | 0.595 | 1.995 | 0.048 |
| **Notes:** Multiple linear regression model included age, gender, education, APOE ε4 positivity, VRS_noDLP,_ and time gap as covariates.  ^a^ F for the model=7.781; adjusted R^2^=0.316. **Abbreviations:** Aβ, beta-amyloid; LDL-C, low density lipoprotein cholesterol; APOE, apolipoprotein; VRS_noDLP_, vascular risk score reflecting vascular risk burden other than dyslipidemia. | | | | | | | |

| **eTable 3.** Multiple linear regression analysis for the interaction between Aβ deposition and other lipids for tau deposition | | | | | | | | |
| --- | --- | --- | --- | --- | --- | --- | --- | --- |
|  |  | |  | Coefficients | | | t | *P* value |
|  |  | |  | B | SE | β |  |  |
| Dependent variable: Tau retention | | | |  |  |  |  |  |
| TC | | Global Aβ retention | | 0.035 | 0.562 | 0.020 | 0.063 | 0.950 |
|  |  | TC | | -0.006 | 0.005 | -0.272 | -10188 | 0.237 |
|  |  | Global Aβ retention x TC^a^ | | 0.005 | 0.003 | 0.568 | 1.491 | 0.139 |
| HDL-C | | Global Aβ retention | | 0.599 | 0.528 | 0.337 | 1.133 | 0.259 |
|  |  | HDL-cholesterol | | -0.005 | 0.015 | -0.084 | -0.349 | 0.727 |
|  |  | Global Aβ retention x HDL-C^b^ | | 0.005 | 0.010 | 0.185 | 0.478 | 0.633 |
| TG | | Global Aβ retention | | 1.291 | 0.319 | 0.726 | 4.042 | 0.000 |
|  |  | TG | | 0.005 | 0.003 | 0.379 | 1.421 | 0.158 |
|  |  | Global Aβ retention x TG^c^ | | -0.004 | 0.002 | -0.501 | -1.605 | 0.111 |
| **Notes:** Multiple linear regression model included age, gender, education, APOE ε4 positivity, and VRS_noDLP_ as covariates.  ^a^ F for the model=8.445; adjusted R^2^=0.311. ^b^ F for the model=8.020; adjusted R^2^=0.341 ^c^ F for the model=8.476; adjusted R^2^=0.312 **Abbreviations:** Aβ, beta-amyloid; TC, total cholesterol, HDL-C, high density lipoprotein cholesterol; TG, triglyceride; APOE, apolipoprotein; VRS_noDLP_, vascular risk score reflecting vascular risk burden other than dyslipidemia. | | | | | | | | |

**eFigure 1.** Multiple linear regression plots showing moderating effects of LDL-C on the relationships between Aβ and tau deposition.


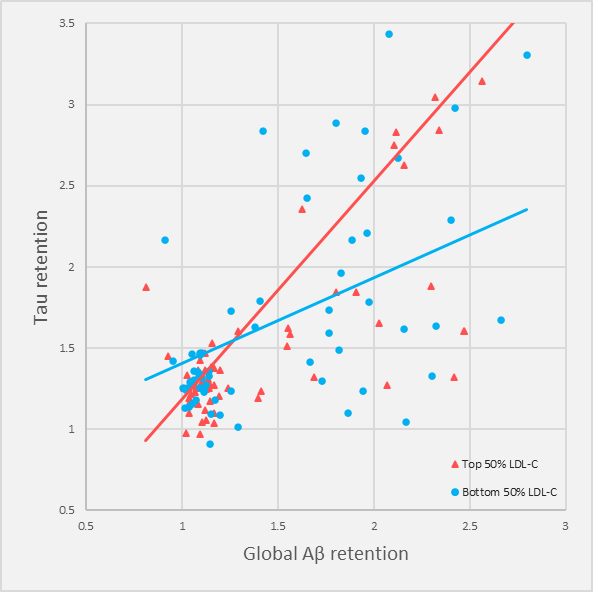


Note: For the purpose of demonstration, participants were divided top 50% LDL-C and the bottom 50% LDL-C subgroups. Multiple linear regression model included Aβ, LDL-cholesterol subgroup, and their interaction term as independent variables; tau retention as dependent variable; and age, gender, education, APOE ε4 positivity, and VRS_noDLP_ as covariates. Statistical significance was observed with the interaction term between Aβ deposition and LDL-C (p < 0.05), as detailed in the manuscript.

Abbreviations: Aβ, beta-amyloid; APOE, apolipoprotein; LDL-C, low density lipoprotein cholesterol; VRS_noDLP,_ vascular risk score reflecting vascular risk burden other than dyslipidemia.
